# Melatonin reverses obesity-induced neurodegeneration through glymphatic restoration

**DOI:** 10.4103/NRR.NRR-D-25-00797

**Published:** 2025-10-30

**Authors:** Bandy Chen

**Affiliations:** Department of Medicine, UC San Diego School of Medicine, La Jolla, CA, USA

Obesity is characterized by both central and peripheral alterations, increasing the risk for neurological and metabolic disorders. Recent evidence indicate that obesity can disrupt the glymphatic system and impair the clearance of cerebrospinal fluid (CSF). This can lead to the build of neurotoxic molecules, potentially explaining obesity’s risk for neurodegeneration and cognitive decline. Given that glymphatic flow is tightly regulated by sleep, and that sleep disturbances are seen in obesity, melatonin emerges as a promising candidate to target glymphatic dysfunction in obesity. In both clinical and preclinical models, exogenous melatonin improves sleep quality and enhances slow-wave sleep, which is the sleep stage when glymphatic clearance is most active. This perspective aims to explore the mechanistic links between obesity and glymphatic dysfunction, highlighting melatonin as a novel therapeutic to mitigate cognitive and neurological consequences associated with obesity through the restoration of the glymphatic system. The mechanistic studies are predominantly based on animal models; therefore, additional human studies that explore the effect of melatonin on glymphatic function are much needed.

**Glymphatic dysfunction as a consequence of obesity:** Obesity is associated with increased risk of developing cognitive decline and neurodegeneration; however, the exact mechanisms involved remain to be elucidated. By adopting a reductional approach that examines specific brain processes altered by obesity, we can construct a more comprehensive understanding of the obese brain. The discovery of the glymphatic system opened a new avenue of research in neuroscience and is becoming a fundamental nexus for understanding the link between brain homeostasis and progression of neurodegenerative diseases. Due to the intimate link between the glymphatic system and other brain processes that are altered in obesity such as neurovascular coupling, cerebral blood flow (CBF), blood–brain barrier (BBB) permeability, myelination, and neuronal activity, it is of great interest to explore whether this novel system that regulates CSF dynamics is also impacted by chronic overnutrition (Chen et al., 2024, 2025a). The following section summarizes recent findings of altered glymphatic activity in different preclinical models of overnutrition.

In mice fed a high-fat diet (HFD) for 10 months, there is accelerated hypothalamic glymphatic activity, characterized by increased influx and elevated aquaporin-4 (AQP4) polarization and density compared to the control group (Delle et al., 2023). This is accompanied by elevated neuroinflammation in both the hypothalamus and the hippocampus. The proposed hypothesis is that the localized increase in glymphatic flow is a compensatory adaptation to obesity-induced changes in the hypothalamus, including higher metabolic demands and nutrient turnover in this specific region. It is quite surprising that there is a lack of global glymphatic flow changes in long-term HFD despite the severe obesity phenotype. One possibility is that glymphatic flow demonstrates phasic responses throughout HFD feeding, where short-term HFD prior to the development of obesity and metabolic dysfunction increases brain-wide glymphatic flow to adapt to elevated nutrient turnover, while long-term HFD with existing obesity and metabolic dysfunction leads to glymphatic dysfunction, AQP4 mislocalization, and vascular remodeling, resulting in a lack of glymphatic flow changes for most regions. This parallels other phasic processes seen in neurodegeneration, such as excitotoxicity seen in early phases of Alzheimer’s disease that transitions into hypoactivity due to neuronal loss. The hypothalamus is a unique region because of its circumventricular nature and its role as a central hub for feeding regulation. This suggests that elevated glymphatic activity may contribute to a self-reinforcing cycle of enhanced food intake and nutrient sensing. This is the first study that explored the glymphatic system after prolonged HFD feeding, hinting at a connection between overnutrition and CSF dynamics to explain obesity-induced neurological complications.

In another long-term HFD mouse model (7 months), there is an overall reduction in glymphatic flow measured by tracer injections into the cisterna magna and corpus striatum (Peng et al., 2025). This is accompanied by reduced AQP4 polarization in the cortex, with a positive correlation between glymphatic clearance activity and AQP4 polarization index. These mice have reduced expression of AQP4 anchoring proteins such as Dag, Snta1, and Dmd, which may explain why AQP4 becomes mislocalized and less effective at mediating fluid transport. Furthermore, clearance of fluorescently labeled amyloid-beta (Aβ) in the hippocampus is impaired (Peng et al., 2025). Disruption in Aβ clearance is exacerbated by TGN-020, an AQP4 inhibitor, whereas TGN-073, an AQP4 enhancer, augments clearance. This suggests that long-term HFD impairs Aβ clearance by suppressing AQP4-mediated glymphatic activity.

To investigate the role of AQP4 during glucose uptake, 18-week HFD mice underwent magnetic resonance imaging to examine CBF and cellular swelling across different brain regions (Tirado-Garcia et al., 2025). Obese mice demonstrate reductions in hippocampal CBF with higher fractional anisotropy and lower radial diffusivity, indicative of gliosis. AQP4 inhibition with TGN-020 reduces glucose-induced microvascular blood flow and cellular swelling, with the most pronounced changes in the hippocampus (Tirado-Garcia et al., 2025). This effect occurred only in the HFD group, likely indicating that AQP4 is functionally upregulated in obesity, where the brain becomes more reliant on AQP4. Given that the inhibitor was delivered via a systemic route, it cannot be assumed that the benefit for the hippocampus is due to an elevated AQP4 expression. Rather, the opposite is likely the case due to the baseline reduction in hippocampal CBF of obese mice. These alterations due to diet or treatment are not found in the cortex, thalamus, or hypothalamus. This finding suggests that the hippocampus is uniquely vulnerable to obesity-related changes in glucose handling and neurovascular dynamics, with AQP4 mediating these effects.

In 16-week HFD mice, there is a downregulation of AQP4 in the caudate putamen, anterior cingulate cortex, and prefrontal cortex (Fu et al., 2025). These regions are part of the frontal-striatal circuit involved in mood regulation and cognition and are highly vulnerable in depression. The reduction in AQP4 coincides with reactive gliosis and upregulation of ciliary neurotrophic factor (CNTF), primarily produced by astrocytes. While it is not statistically significant, high-dose CNTF slightly reduces AQP4 mRNA expression in striatal astrocytes *in vitro* (Fu et al., 2025). This hints that CNTF may downregulate AQP4 expression or influence its localization, particularly under inflammatory conditions. Furthermore, increased expression of CNTF in the frontal-striatal circuit is associated with loss of oligodendrocytes and myelin basic protein reduction in the corpus callosum of HFD mice. *In vitro*, high concentrations of CNTF impair oligodendrocyte differentiation and reduce expression of key myelination genes such as Myrf, PLP1, and myelin basic protein (Fu et al., 2025). Future studies aim at elucidating the mechanistic links between CNTF and downstream AQP4 polarization and oligodendrocyte differentiation will be fundamental in understanding the link between glymphatic dysfunction and demyelination in the context of obesity.

Lastly, there is elevated immune cell infiltration in the meninges with elevated C-reactive protein levels in the blood, suggesting that peripheral inflammation spreads into the brain and disrupts meningeal lymphatic drainage (Fu et al., 2025). The lack of drainage can impede CSF outflow and result in the accumulation of neurotoxic molecules. This highlights the meninges as a key site of early immune dysregulation in obesity. Being closely linked to the glymphatic system, further examination of the meninges in the context of obesity could elucidate key mechanisms underlying obesity-induced neurological dysfunction.

In summary, alterations in glymphatic function in the context of obesity exhibit spatiotemporal specificity. These changes are not uniform across the brain but instead manifest differentially over time and in anatomically distinct regions. This leads to a speculative yet plausible hypothesis where regions that are near circumventricular organs with nutrient sensing capability, such as the hypothalamus, are more capable to adapt to nutrient shifts, while higher-order regions, such as the cortex and hippocampus, are less plastic and more prone to degeneration. To overcome the lack of spatiotemporal resolution, developing a glymphatic clearance atlas across different phases of overnutrition can help identify the phasic responses of glymphatic activity and allow interventions to be precisely timed and localized to mitigate dysfunction.

**Melatonin in mitigating obesity-induced neurological complications:** Melatonin is a neurohormone secreted mainly by the pineal gland and exerts pleotropic effects in the brain, including circadian modulation, antioxidant defense, neuroimmune regulation, synaptic plasticity, and modulation of neurogenesis. These processes are dysregulated with reduced endogenous melatonin levels in the obese brain. Recent data demonstrate the potential of utilizing exogenous melatonin to improve both neurological and metabolic dysfunctions seen in obesity. The following section explores the effect of melatonin on obesity-induced neurological complications and dives into the possible mechanisms that mediate these effects.

In aged rats exposed to 10 months of HFD, melatonin treatment attenuates peripheral insulin resistance and reduces hippocampal neuroinflammation and oxidative stress (Xu et al., 2019). In addition, melatonin also prevents HFD-induced Aβ accumulation and tau phosphorylation and preserves cholinergic neuronal activity. The anti-inflammatory and neuroprotective effects result in improved functional and behavioral outcomes such as mitigating cognitive decline and hippocampal long-term potentiation (Xu et al., 2019). It is proposed that these downstream effects are mediated through the effect of melatonin on brain insulin signaling. In a type 2 diabetes mellitus mouse model, melatonin demonstrates protective effect against both metabolic dysfunctions and neuropsychiatric injuries (Gao et al., 2024). Melatonin improves diabetes syndromes by lowering blood glucose, cholesterol, and triglyceride levels, while improving insulin levels and liver and pancreas function. Interestingly, these diabetic mice exhibit reduced depression- and anxiety-like behaviors measured by tail suspension and forced swim tests and enhanced memory and learning in the Y-maze and novel object recognition tests (Gao et al., 2024). The central effects of melatonin are mediated through the restoration of the hippocampus, including the quiescence of inflammatory glial cells, reduction in neurotoxic molecules such as Aβ and phosphorylated tau, elevation in neurotrophic molecules such as brain-derived neurotrophic factor, synapsin I, and synaptotagmin I, and restoration of melatonin receptor and circadian rhythm genes (Gao et al., 2024). These findings indicate that melatonin can mitigate metabolically induced brain dysfunction by improving insulin signaling and halting neurodegeneration.

The central effects of melatonin have the potential to extend across generations, influencing neurodevelopmental and metabolic outcomes in offspring. In maternal HFD (10 weeks of HFD for the mother and 7–11 weeks of HFD for the offspring), both the mother and the offspring demonstrate elevated neuroinflammation marked by increased tumor necrosis factor-α, interleukin-1β, and cyclooxygenase-2, elevated expression of apoptotic markers such as cleaved caspase-3, poly [ADP-ribose] polymerase 1 and Bax, and greater synaptic dysfunction due to reduced levels of postsynaptic density 95 and synaptophysin (Jan et al., 2024). Both the mother and offspring have elevated BACE-1 and Aβ levels indicative of amyloidogenic burden and impaired short- and long-term memory in Y-maze and Morris Water maze tests. Co-administration of melatonin with HFD ameliorates neuronal apoptosis and amyloid burden, preserves synaptic proteins, and restores central insulin signaling (Jan et al., 2024). This results in improved short- and long-term memory in both the mother and offspring. These neuroprotective effects are proposed to be mediated through SIRT-1 dependent activation of the nuclear factor erythroid 2-related factor 2/Heme oxygenase-1 pathway (Jan et al., 2024). Maternal HFD for 14 weeks reprograms the hypothalamic circuitry of the offspring toward hyperphagia and leptin resistance, which increases the risk of cognitive decline and metabolic disruption. Treatment with melatonin during gestation and lactation ameliorates hypothalamic inflammation, increases anorexigenic POMC expression, and decreases orexigenic NPY expression (Nagagata et al., 2024). Interestingly, the beneficial effects extend to the periphery with increased conversion of subcutaneous white adipose tissue to the beige phenotype and elevated thermogenic capacity of interscapular brown adipose tissue (Nagagata et al., 2024). Whether this is mediated by the direct action of melatonin on the adipose tissue or an indirect top-down effect remains to be elucidated. Overall, these findings indicate that melatonin can counteract HFD-induced metabolic programming, resulting in decreased food intake and body weight and improved cognitive function.

Due to significant impact of obesity on the cerebral vasculature and the glymphatic system, these alterations heighten the risk for cerebrovascular events, such as ischemic stroke, and impair the ability of the brain to recover following such injuries. Recent evidence suggests that melatonin may counteract the predisposing factors of obesity for stroke and enhance neurological recovery following cerebrovascular injury. In 16-week HFD mice subjected to cerebral ischemia and reperfusion injury, melatonin administration at reperfusion reduces infarct volume and neurological deficits and preserves neuronal morphology and BBB integrity in the peri-infarct cortex (Yawoot et al., 2023). Furthermore, melatonin treatment suppresses glial activation and inhibits both pyroptosis and necroptosis pathways. This is mediated by the downregulation of high mobility group box 1/Toll-like receptor 4/nuclear factor-κB axis, which disrupts the inflammatory feedback loop. While the mechanisms through which melatonin counteracts obesity-induced neurological complication requires further clarification, its modulation of the glymphatic system emerges as a plausible and promising candidate.

**Figure 1:**
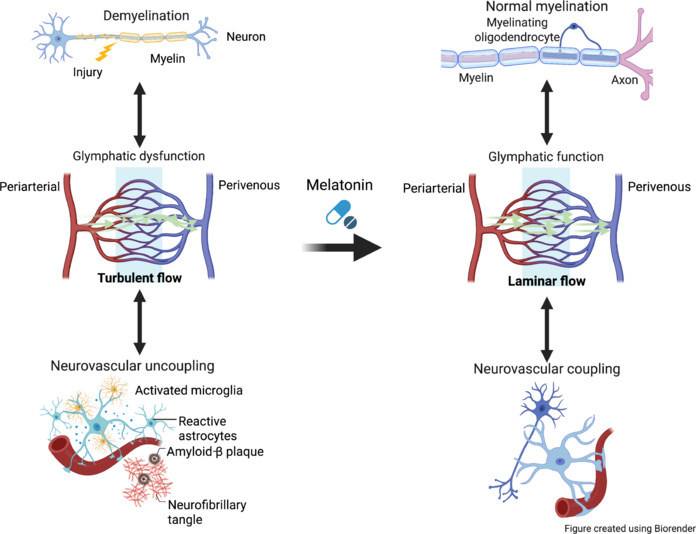
Melatonin facilitates recovery of glymphatic flow. Due to the intimate link between the glymphatic system and other brain processes such as myelination and neurovascular coupling, dysfunction in the glymphatic system is associated with demyelination and neurovascular uncoupling. This results in a domino effect of neurodegeneration. Melatonin improves glymphatic function by enhancing sleep quality, increasing aquaporin-4 polarization, and reducing neuroinflammation. Improved glymphatic function results in an enhanced local milieu, leading to a cascade of neural repair. These mechanisms shed light on the broad neurological effects of melatonin. Created with BioRender.com.

**Melatonin as a key regulator of glymphatic flow:** Given that glymphatic function is highly sleep-dependent, chronobiotic properties of melatonin hint at the potential to regulate glymphatic function. Recent studies indicate that exogenous melatonin not only improves sleep architecture but also enhances glymphatic flow and neurovascular coupling (**Figure 1**). This provides a mechanistic basis for how melatonin rewires the obese brain. The following section outlines the various mechanisms by which melatonin modulates the glymphatic system.

In the chronic unpredictable mild stress mouse model of depression, there is impaired glymphatic function throughout the sleep-wake cycle due to astrocytic loss and disrupted circadian regulation of AQP4 expression in perivascular astrocytes (Yao et al., 2023). This is accompanied by reduced total sleep time and non-rapid eye movement sleep, primarily due to increased sleep fragmentation during the light phase. Treatment with melatonin restores glymphatic function and APQ4 polarization and improves sleep structure and depressive behaviors (Yao et al., 2023). The group identified a novel mechanism through which melatonin alleviates depressive symptoms by modulating the expression of the circadian protein Per2, which is negatively correlated with AQP4 polarization (Yao et al., 2023). In a modified rotating rod sleep restriction mouse model, there are short-term memory deficits and AQP4 mislocalization in the hippocampus (Sun et al., 2025). These impairments are reversed with melatonin treatment with a dose-dependent effect. Melatonin enhances glymphatic flow, reduces Aβ and phosphorylated tau levels, and ameliorates glial reactivity in the hippocampus (Sun et al., 2025). Interestingly, protective effects of melatonin against chronic sleep restriction are abolished in AQP4 knockout mice, indicating that proper AQP4 polarization is essential for neuroprotective role of melatonin (Sun et al., 2025). At the mechanistic level, melatonin activates the vitamin D receptor, leading to increased expression of dystrobrevin alpha, a critical component of the dystrophin-associated complex. This cascade restores AQP4 polarization during chronic sleep restriction conditions (Sun et al., 2025). These findings indicate that the neuroprotective effects of melatonin against sleep deprivation-induced brain dysfunction depend on AQP4-mediated glymphatic clearance.

In a mouse model of intracerebral hemorrhage, there are cognitive and behavioral impairments, elevated brain edema, and reduced CBF (Chen et al., 2025). Melatonin treatment alleviates these impairments, while luzindole (a melatonin antagonist) blocks these therapeutic effects. Furthermore, melatonin augments glymphatic flow and reduces BBB permeability with reduction in reactive astrocytes and improvement in AQP4 polarization (Chen et al., 2025). In rats with permanent middle cerebral artery occlusion, melatonin treatment results in significant reduction in infarct volume and improvement in neurobehavioral outcome (Lee et al., 2024). This is coupled with decreased BBB permeability and brain edema, along with lower expression of AQP4 and MMP-9 proteins and enhanced maintenance of ZO-1 protein integrity. It is proposed that the neuroprotective effect of melatonin after cerebral ischemia and reperfusion injury is mediated through the phosphoinositide 3-kinase/AKT/nuclear factor erythroid 2-related factor 2 pathway (Liu et al., 2025). Administration of phospho-phosphoinositide 3-kinase inhibitor Ly294002 after cerebral ischemia and reperfusion injury results in elevated AQP4, indicating this pathway is a key downstream regulator of therapeutic effects of melatonin. Collectively, these findings highlight that melatonin can improve glymphatic function through AQP4 polarization, while also inhibiting post-injury increases in AQP4 activation and cytotoxic edema.

**Conclusions:** Melatonin improves obesity-induced neurological complications through a variety of interrelated mechanisms. One promising mechanism involves its regulation of the glymphatic system for removing metabolic waste, distributing nutrients, and maintaining extracellular fluid balance. Glymphatic function may be restored through multiple routes, including enhancing sleep quality and architecture, regulating AQP4 expression and polarization, reducing neuroinflammation, and stabilizing cerebrovascular function. These effects position melatonin as a multifunctional agent to mitigate the neurocognitive consequences of obesity. The benefits also extend to the periphery, with melatonin improving metabolic dysfunction, such as enhancing insulin sensitivity and glucose metabolism, regulating adipose tissue function, and restoring lipid profile. While this perspective explores the role of melatonin in obesity, these therapeutic effects may also apply to other neurometabolic diseases such as Alzheimer’s disease, where both the brain and the body worsen through disease progression.

**Additional file:** *Open peer review report 1.*

OPEN PEER REVIEW REPORT 1
