# Supplementary material for: Melatonin reverses obesity-induced neurodegeneration through glymphatic restoration
Source: Neural Regen Res. 2025 Oct 30;21(7):2956–7. doi: 10.4103/NRR.NRR-D-25-00797 (PMC13378919; doi:10.4103/NRR.NRR-D-25-00797)
Supplement: Supplementary file 1 [file NRR-21-2956_Suppl1.pdf]

OPEN PEER REVIEW REPORT 1

Name of journal: Neural Regeneration Research
Manuscript NO: NRR-D-25-00797
Title: Melatonin as a therapeutic modulator of glymphatic dysfunction in obesity
Reviewer's Name: Roberto C Salgado-Delgado
Reviewer's country: Mexico

COMMENTS TO AUTHORS

This article examines the impact of obesity on the glymphatic system, a crucial mechanism for waste removal from the brain, and proposes melatonin as a potential therapeutic intervention. The mechanisms by which obesity may impair glymphatic function, contributing to neurological and cognitive problems, are discussed. Research suggests that melatonin may improve sleep quality and restore glymphatic activity, which in turn could mitigate the neurological consequences associated with weight gain. Furthermore, the text examines the broader protective effects of melatonin on metabolic and brain health in various preclinical models of obesity and related conditions. Emphasis is placed on melatonin's ability to regulate the expression and polarization of aquaporin-4 (AQP4), a key component of the glymphatic system.

The article it aims to explore a topic, propose hypotheses, and suggest future research directions rather than presenting new experimental data or a systematic review. This type of article generally allows for a more speculative and forward-looking tone.

-The introduction clearly and effectively states the article's aim from the outset. It establishes the connection between obesity and glymphatic dysfunction, highlighting that obesity can disrupt the glymphatic system, leading to the accumulation of neurotoxic molecules and an increased risk for neurodegeneration and cognitive decline. The article then positions melatonin as a "novel therapeutic" to mitigate these consequences by restoring the glymphatic system. This immediate clarity helps the reader understand the core argument.

-The writing excels at detailing the proposed mechanisms through which melatonin might exert its effects. For instance, it explains how melatonin improves sleep quality and enhances slow-wave sleep, which is crucial for glymphatic clearance. It also delves into more complex cellular mechanisms such as the regulation of aquaporin-4 (AQP4) expression and polarization by melatonin, including the activation of the vitamin D receptor leading to increased Dystrobrevin Alpha and restored AQP4 polarization. Furthermore, the article describes how melatonin reduces neuroinflammation and glial reactivity by downregulating the HMGB1/TLR4/NF-κB axis and stabilizes cerebrovascular function and blood-brain barrier (BBB) permeability.

The writing demonstrates a nuanced understanding of glymphatic dysfunction in obesity, noting that alterations "exhibit spatiotemporal specificity" and are "not uniform across the brain". It provides examples of seemingly contradictory findings, such as accelerated hypothalamic glymphatic activity in long-term HFD models, which is hypothesized as a "compensatory adaptation", versus an overall reduction in glymphatic flow in other long-term HFD models. This sophisticated approach enriches the discussion and shows a deep engagement with the current literature.

The writing is well-supported by numerous recent studies, with publication years like 2023, 2024, and 2025 frequently cited. This indicates that the authors are up to date with the latest advancements and

empirical evidence in the field, strengthening the article's claims.

CRITICISMS AND AREAS FOR IMPROVEMENT:

1. A significant portion of the detailed mechanistic explanations and findings are drawn from animal models (mice and rats). While the introduction mentions melatonin improving sleep quality in "clinical and preclinical models", the deep dive into glymphatic mechanisms is predominantly based on animal studies. The writing does not explicitly discuss the limitations of directly generalizing these findings to human obesity and neurological complications, which is a common challenge in translational research.
2. The article's honesty in highlighting areas that "require further clarification" or "remain to be elucidated" is a scientific strength, but also implicitly points to current gaps in knowledge within the field. For example, it states, "Whether this [melatonin's beneficial effects in the periphery] is mediated by melatonin's direct action on the adipose tissue or an indirect top-down effect remains to be elucidated". Similarly, it mentions that "the mechanisms through which melatonin counteracts obesity-induced neurological complication requires further clarification". While transparent, this indicates that some proposed pathways are still hypothetical and lack full empirical validation.
3. The article itself identifies the need to "develop a glymphatic clearance atlas across different phases of overnutrition" to enable precise, timed, and localized interventions. The current absence of such an atlas means that our understanding of the precise spatiotemporal specificity of glymphatic dysfunction in obesity is still evolving, which could affect the precision of future interventions discussed in the piece.
4. As a "Perspectives" article, the writing naturally adopts a proactive and optimistic tone about melatonin's potential. However, it does not delve into potential adverse effects of melatonin, optimal human dosages, drug interactions, or individual variability in response in the context of human obesity. While not a flaw for this article type, these are crucial considerations for clinical translation that are not addressed within the provided text.
5. Some statements are presented as plausible suggestions rather than definitively proven mechanisms. For instance, "This suggests that elevated glymphatic activity may contribute to a self-reinforcing cycle of enhanced food intake and nutrient sensing". While a compelling hypothesis, the exact empirical evidence for this "self-reinforcing cycle" and its direct contribution to pathology is not detailed with specific studies within the provided excerpt.

In summary, the writing in "Melatonin as a therapeutic modulator of glymphatic dysfunction in obesity" is insightful, well-structured, and rich in scientific detail, effectively fulfilling its role by proposing a promising therapeutic avenue. The main criticisms relate to the inherent challenges of translating preclinical findings to human applications and the acknowledgment of ongoing research needs, which the article transparently communicates itself.
